# Syncope with Surprise: An Unexpected Finding of Huge Gastric Diverticulum

**DOI:** 10.1155/2016/1941293

**Published:** 2016-05-26

**Authors:** Mauro Podda, Jenny Atzeni, Antonio Messina Campanella, Alessandra Saba, Adolfo Pisanu

**Affiliations:** Department of Surgical Science, General, Emergency and Laparoscopic Surgery, University of Cagliari, Blocco G, 09042 Monserrato, Italy

## Abstract

A gastric diverticulum is a pouch protruding from the gastric wall. The vague long clinical history ranging between dyspepsia, postprandial fullness, and upper gastrointestinal bleeding makes this condition a diagnostic challenge. We present a case of large gastric diverticulum that has been diagnosed during clinical investigations for suspected cardiovascular issues in a patient admitted at the medical ward for syncope. A 51-year-old man presented to the medical department due to a syncopal episode occurring while he was resting on the beach after having his lunch, with concomitant vague epimesogastric gravative pain without any other symptom. A diagnosis of neuromediated syncopal episode was made by the cardiologist. Due to the referred epimesogastric pain, an abdominal ultrasound scan was carried out, showing perisplenic fluid. A CT scan of the abdomen was performed to exclude splenic lesions. The CT scan revealed a large diverticulum protruding from the gastric fundus. The upper gastrointestinal endoscopy visualized a large diverticular neck situated in the posterior wall of the gastric fundus, partially filled by undigested food. The patient underwent surgery, with an uneventful postoperative course. Histologic examination showed a full-thickness stomach specimen, indicative of a congenital diverticulum. At the 2nd month of follow-up, the patient was asymptomatic.

## 1. Introduction

A gastric diverticulum (GD) is a pouch protruding from the gastric wall, first described by Moebius in 1661 and later by Roax in 1774 [1]. Although it is the least common diverticulum of the gastrointestinal tract, GD has similar characteristics to duodenal, jejunal, and colonic diverticula [2]. It is a rare and uncommon clinical condition, with a prevalence of 0.03–0.1% in contrast upper gastrointestinal radiographs, 0.01–0.1% in upper gastrointestinal endoscopy, and 0.03–0.3% in autoptical reports [3]. Incidence is equally distributed between males and females. Most symptomatic diverticula are found in patients between 20 and 60 years of age [4]. Typical diverticula are 1–3 cm in diameter, but larger types can occur [5]. The lack of pathognomonic symptoms and the vague long clinical history ranging between dyspepsia, postprandial fullness, and upper gastrointestinal bleeding make this condition a diagnostic challenge for physicians and surgeons [6]. We present the case of a large GD that has been diagnosed during clinical investigations for suspected cardiovascular issues in a patient admitted at the medical ward for syncope.

## 2. Case Report

**Figure 1:**
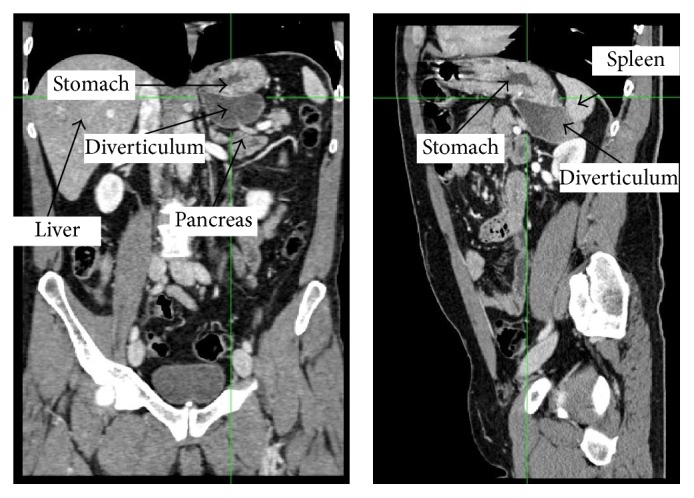
Arterial phase CT scan (frontal and sagittal planes) showing a large diverticulum of the size of 52 × 68 × 72 millimeters protruding from the gastric fundus, with fluid content. The retroperitoneal location of the pouch is well visible in the sagittal scan, as well as its tight adhesions with the inferomedial surface of the spleen, the ipsilateral adrenal gland, and the upper posterior surface of the body and tail of the pancreas.

**Figure 2:**
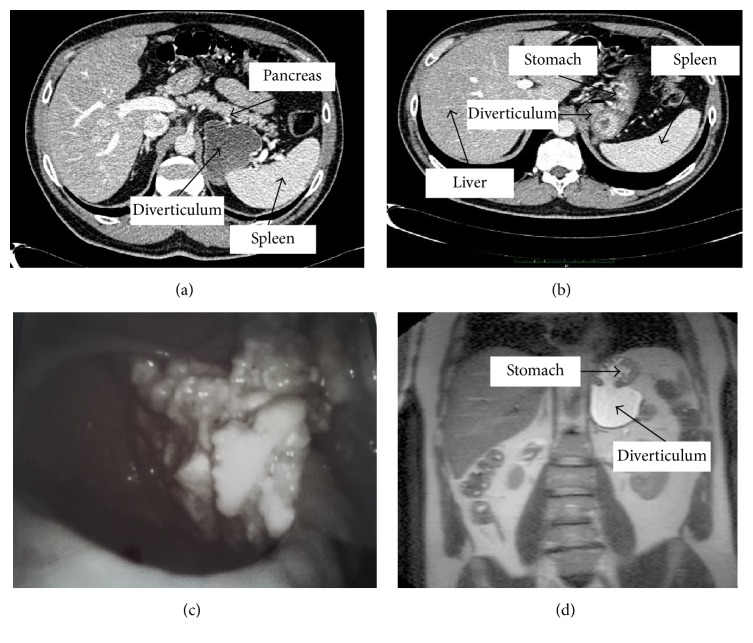
Arterial phase CT scan, transverse planes (a-b). Endoscopic image of a large diverticular neck (30 × 20 millimeters) situated in the posterior wall of the gastric fundus, with the pouch partially filled by undigested food (c). T2-weighted MRI image (d).

A 51-year-old Caucasian policeman was admitted at the medical department of the University of Cagliari Hospital (Italy) due to a syncopal episode occurring while he was resting on the beach after eating his lunch, with concomitant vague epimesogastric gravative pain without any other symptoms. In the anamnesis he reported a previous similar episode, occurring 10 years earlier, during a walk in a shopping center after having a carbonated beverage (cola). For this reason, he underwent cardiovascular and neurological investigations. The electrocardiogram, echocardiogram, and stress test on the treadmill were unremarkable. The head-up tilt-table test reproduced the original symptoms, with objective evidence of a sudden drop in blood pressure without a decrease in heart rate. Therefore, a final diagnosis of neuromediated syncopal episode was carried out. Due to the referred epimesogastric pain, an abdominal US scan was carried out, showing a small amount of perisplenic fluid. A CT scan and an MRI scan of the abdomen were performed in order to exclude splenic lesions. The CT scan revealed a large diverticulum of the size of 52 × 68 × 72 millimeters protruding from the gastric fundus showing tight adhesions with the inferomedial surface of the spleen, the ipsilateral adrenal gland, and the upper posterior surface of the body and tail of the pancreas (Figures 1 and 2). The patient was therefore referred to our surgical team. When a deeper anamnesis was carried out, the patient reported a two-year symptomatology characterized by recurrent dyspepsia, postprandial fullness, and frequent burping with fetor ex ore.

**Figure 3:**
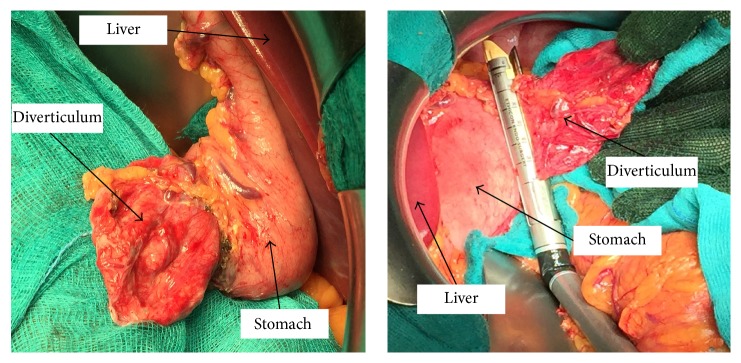
Intraoperative view. The gastrocolic ligament and the short gastric vessels have been released and the stomach is rotated to expose the superior-posterior wall. The adhesions between the diverticulum and the posterior surface of the pancreatic body have been dissected. Exposure of the neck of the diverticulum for preparation of diverticulectomy and resection of the neck with the linear stapler.

On physical examination the hemodynamics were stable, and the patient had a mild epigastric tenderness. Abdominal and thoracic examination was otherwise unremarkable. Blood tests and chest and abdominal X-rays were normal. The upper gastrointestinal endoscopy visualized a large diverticular neck (30 × 20 millimeters) situated in the posterior wall of the gastric fundus. After further exploration, a pouch partially filled by undigested food was discovered (Figure 2). Due to the large volume of the diverticulum and its retroperitoneal location patient underwent open surgery. The resection of the gastric diverticulum was performed using an Echelon Flex Powered Endopath 60 mm gold reload (Ethicon Endo-Surgery, Cincinnati, Ohio, USA). The stapler line was oversewn by a running suture with resorbable coated polyclacin 2-0 suture (Vicryl*™*, Ethicon) (Figure 3). The patient had an uneventful postoperative course. On postoperative day one, a barium study showed normal anatomy and no leaks. He was placed on a fluid diet on postoperative day one and regular diet on postoperative day three. The patient was discharged on postoperative day five. Histologic examination showed a full-thickness stomach specimen, indicative of a congenital diverticulum, with slight chronic inflammation and diffuse hyperplasia and hypertrophy of the oxyntic cells. At the 2nd month of follow-up appointment, both epigastric pain and halitosis had disappeared.

## 3. Discussion

According to the hypothesis suggested by Schmidt and Walters, GD can by classified into congenital and acquired types, with congenital types being more common [5, 7, 8]. As reported in the literature, congenital gastric diverticula are true diverticula, mostly located in the posterior wall of the fundus, 2 cm below the oesophagogastric junction and 3 cm from the lesser curve (70%). They contain all layers of the gastric wall, and it is believed that they occur as a result of splitting of the longitudinal muscular fibers at the cardia level, leaving only circular muscle fibers in the gastric wall through which a diverticulum can develop during the fetal period [9, 10]. Conversely, false diverticula are acquired, less common and typically located in the antrum. Acquired diverticula can develop either with a traction or pulsion mechanism and usually present with a background history of chronic gastrointestinal inflammatory disease, such as peptic ulcer, pancreatitis, malignancy, or gastric outlet obstruction [5, 8, 11, 12]. The development of congenital GD within the retroperitoneal space can be explained by the analysis of the embryogenesis of the stomach in the period between the 20th and the 50th day of gestation. At this time, a 90° rotation of the stomach, together with the duodenum, the pancreas, and the dorsal mesentery, occurs and a diverticulum of the posterior wall of the gastric fundus could hypothetically herniate through an area of dorsal mesentery before its fusion with the left posterior body wall. Therefore, with further extension, the diverticulum could project posterior to the pancreas [6, 13].

Being a rare and mostly asymptomatic condition, a high clinical index of suspicion is needed to diagnose and effectively manage patients with GD [6]. When symptoms occur, they can vary and imitate those of other common upper gastrointestinal disorders. Indeed, the most common complaint reported by symptomatic patients is a vague upper abdominal pain (18–30%), possibly due to food retention inside the diverticulum and subsequent distension of the pouch [14]. Other complaints include vomiting, vague sensation of fullness or discomfort in the upper abdomen, dysphagia, eructation, and halitosis, which could be explained by the bacterial overgrowth on the food retained inside the GD [15, 16]. Moreover, this condition can evolve to dramatic scenarios, such as massive bleeding or perforation following the digestive process of the retained food by gastric juices. This causes possible ulceration of the mucosa or complicated diverticulitis [17]. Two cases of invasion with adenocarcinoma in gastric diverticulum were also reported in the literature [18, 19].

GD is considered as a great mime: Palmer found that in 30 of 49 symptomatic patients with GD the symptomatology was ascribable to other gastrointestinal diseases [20].

Our patient presented with postprandial loss of consciousness, with no remarkable cardiogenic problem proved. Carbonated beverages ingestion, as reported by the patient before his first syncopal attack, and the presence of eructation have already been involved with the triggering in some cases of neuromediated syncope [21]. Moreover, many gastroesophageal diseases such as achalasia, diffuse oesophageal spasm, hiatal hernia, and diverticulum have been reported to be the trigger of syncopal episodes called “Swallow Syncope,” which is defined as a dysautonomic syndrome associated with intense vagal afferent activation [21–25].

In the majority of cases GD are incidental findings during the investigation of their common symptoms. The condition can be diagnosed by endoscopic or radiological examinations. Upper gastrointestinal contrast radiographic studies, as well as oesophagogastroduodenoscopy, are the most performed diagnostic investigations. However, it is worth emphasizing that, especially in case of diverticulum with a narrow neck, they can give false negative results. Indeed, as reported by Palmer in his review, 5% of GD are missed during contrast radiographic study [20].

Upper gastrointestinal endoscopy is the gold standard investigation to achieve a precise diagnosis. It useful not only to confirm the location and the size of the pouch, but also to perform a biopsy if a concurrent pathology is found. This diagnostic tool may be able to reproduce symptoms with distension of the diverticulum, indicating which patients would benefit from a resection [5, 12].

More authors have reported their experience on the use of CT scan as part of the diagnostic work-up for patients with gastric diverticulum. However, CT and/or MRI findings in GD have been published only as case reports in the literature [3, 26–28]. CT and MRI scans can provide a complete overview on the relationships between the diverticulum and the pancreatic gland, the spleen, and the left adrenal gland. These diagnostic tools are necessary during the planning of the surgical strategy, as proven by our case report.

The appropriate treatment for both symptomatic and asymptomatic GD is still a matter of debate. It is well documented that there is no specific therapeutic strategy for asymptomatic diverticula [10, 20].

Although Proton Pump Inhibitors (PPIs) have been suggested to alleviate the vague symptoms of gastric diverticulum, this treatment is not able to resolve the underlying pathology. Endoscopy can play an important role in the management of active upper gastrointestinal bleeding due to GD, as reported by Chen et al. [2]. Surgical resection is the recommended approach when the diverticulum is large (>4 cm in diameter), patients are still symptomatic after PPIs administration, and complications such as bleeding, perforation, or suspicion of malignancy occur [6]. Both open and laparoscopic resections achieve good results. Since Fine in 1998 described the first successful laparoscopic resection of a large proximal gastric diverticulum, this approach is now considered safe and feasible, with excellent outcomes [29]. Access to the retrogastric space by dividing the gastrocolic/gastrosplenic ligament is often easier to achieve laparoscopically in experienced hands, although in other cases the minimally invasive approach can be challenging because the diverticulum is often collapsed, hidden in the splenic bed, or deeply adherent to the posterior surface of the pancreas [6, 9, 20, 30]. Rate of success is very high, with over two-thirds of patients remaining symptom-free after surgical resection [20].

Although GD remains a rarity in the etiology of abdominal pain, a high index of suspicion should be kept in mind for patients with a long history of vague upper abdominal pain and dyspepsia, especially when not subsided with PPIs and when all other more common diseases, such as gastritis and gastric cancer, have been ruled out.

## Abbreviations

GD: Gastric diverticulum
CT: Computed tomography
MRI: Magnetic resonance imaging
US: Ultrasound
PPI: Proton Pump Inhibitors.
